# Light weight meshes in incisional hernia repair

**DOI:** 10.4103/0972-9941.27722

**Published:** 2006-09

**Authors:** Volker Schumpelick, Uwe Klinge, Raphael Rosch, Karsten Junge

**Affiliations:** Department of Surgery, University Hospital Aachen, Germany

**Keywords:** Incisional hernia, mesh, polypropylene, recurrence

## Abstract

Incisional hernias remain one of the most common surgical complications with a long-term incidence of 10–20%. Increasing evidence of impaired wound healing in these patients supports routine use of an open prefascial, retromuscular mesh repair. Basic pathophysiologic principles dictate that for a successful long-term outcome and prevention of recurrence, a wide overlap underneath healthy tissue is required. Particularly in the neighborhood of osseous structures, only retromuscular placement allows sufficient subduction of the mesh by healthy tissue of at least 5 cm in all directions. Preparation must take into account the special anatomic features of the abdominal wall, especially in the area of the Linea alba and Linea semilunaris. Polypropylene is the material widely used for open mesh repair. New developments have led to low-weight, large-pore polypropylene prostheses, which are adjusted to the physiological requirements of the abdominal wall and permit proper tissue integration. These meshes provide the possibility of forming a scar net instead of a stiff scar plate and therefore help to avoid former known mesh complications.

It is generally not the hernia itself or the technique required for repair but the patients and their comorbid conditions that generate difficulty during the management of this condition. Patients who develop an incisional hernia after suture closure of the fascia following laparotomy form a special group of individuals with specific pathophysiologic abnormalities. Technical failures are of minor importance and account for less than half of the wound failures after 1 year or later. Several attempts have been made to identify risk factors, but there is no general agreement on which of these factors is essential. Neither the suture material nor the suture technique is absolute. At best, the use of continuous, long-term absorbable or nonabsorbable sutures can decrease the incidence of incisional hernias when applied in a 4:1 ratio of suture length to wound length.[1,2] However, even with the best technique, an incidence of at least 10% must be accepted as the long-term result.[2,3]

## PATHOPHYSIOLOGY

For decades the incisional hernia was hypothesized to be caused mainly by technical problems with suture technique. Consequently, correction of this problem was undertaken by a repeat but more meticulous suture repair with a variety of configurations to prevent re-recurrence. Additional doubling of the fascia to reinforce the abdominal wall was performed in some cases. Whereas the intraoperative aspects were ostensibly satisfying, the long-term results were disappointing. Recurrence rates of 50% after suture repair of an incisional hernia were reproduced in several studies.[4,5] It was the introduction of mesh by Usher *et al* in 1958 that opened a new era.[6,7] Reinforcement of the abdominal wall with strong polyester or polypropylene nets produced a resilient scar-mesh compound that prevented recurrences through the mesh. Indeed, recurrences through mesh are still a rarity. In accordance with the widespread use of mesh, several personal series reported excellent results, with recurrence rates of far less than 10%. Examination of the literature shows that the results were independent of mesh type and operative technique.[3,8] This early euphoria has been recently clouded by the results of the only randomized controlled trial that has compared mesh and suture repair.[9,10] In this Dutch trial,[9,10] a mesh was applied in a prefascial, retromuscular position with an overlap of 2 to 4 cm. After 3 years, there were 43% recurrences in the suture group, as expected. However, the authors found 24% recurrence in the mesh group as well. Interestingly, the results were not affected by the size of the hernia. Furthermore, in 2003 a retrospective population-based cohort study by Flum *et al* analyzed data from 10,822 patients operated on incisional hernias by either suture or unspecified mesh repair.[11] Within 5 years, 14% of the patients underwent at least one subsequent re-repair after suture repair compared to 11% after mesh repair. First, both studies clearly revealed a reduced recurrence rate after mesh implantation at any time point. The lower rate seen by Flum *et al* might be due to the fact that several patients with a relapse have not undergone reoperation. Nevertheless, the most striking fact is that both studies unexpectedly found a constantly rising incidence of hernia recurrence over the years, not only in the suture group but in the mesh group as well. Over a decade, this recurrence rate shows an almost linear curve. Comparing suture to mesh, the implants only seem to delay the recurrence for 2[11] or 4[10] years respectively. These data substantiate that the development of a recurrence in an incisional hernia repair is not primarily a technical one. As a consequence, the only plausible explanation is that the phenomenon is a biologic one. Recently, molecular biologic investigations have proven the theory of disturbed composition of the extracellular matrix in patients with recurrent hernia. In particular, there is a decreased ratio of collagen types I and III.[12,13] Furthermore, *in situ* dysfunction in fibroblasts has been found, indicating a primary malfunction of the cells independent of their local environment. This wound-healing perspective of incisional hernia formation explains the following observations: an incisional hernia manifests after considerable delay following the previous operation, the cumulative incidence shows a linear time course, repetition of a previously inadequate technique frequently fails, reinforcement of the entire scar is advisable irrespective of the intraoperative evidence, patients with an aortic aneurysm or a proven defect in collagen metabolism exhibit an increased incidence of incisional hernias, the use of absorbable materials leads to a high relapse rate and the success of a mesh repair depends on the extent of overlap.

Reinforcement of the closed hernial gap by mesh is based on the concept of in growth of fibrous tissue into prosthetic material, forming a scar-mesh compound. Although the intensity of the scar formation is influenced by the amount of material, its quality is not improved.[14] As a consequence, mesh fixation by fibrosis cannot prevent recurrence unless a wide overlap underneath healthy tissue can be achieved. Although such data are not yet published, it may be hypothesized that the width of the overlap correlates with the duration of the delay. However, clinical experience with all techniques developed in recent years has uniformly shown a trend towards utilizing larger prostheses. In conclusion, a defective process of wound healing should be assumed in patients suffering from incisional hernia. Rare exceptions refer to patients with a traumatic defect or an obvious technical fault (e.g., unclosed trocar incisions or broken suture material). Consequently, this principle must be carefully considered regarding technique and performance.

## CLINICAL PRESENTATION AND DIAGNOSTIC METHODS

**Figure 1:**
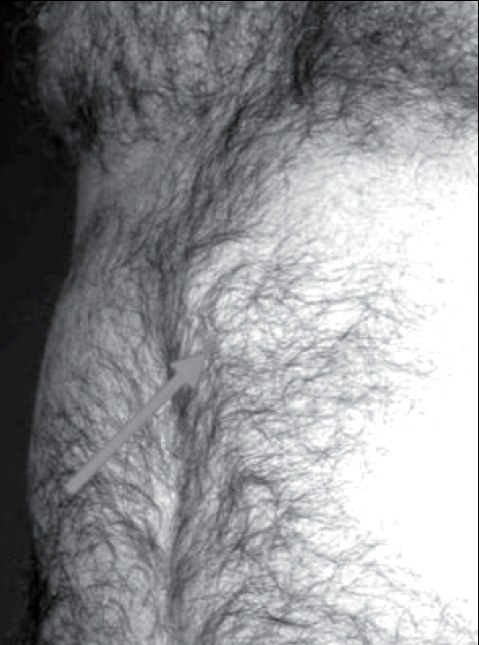
Incisional hernia formation following midline laparotomy

By definition, an incisional hernia represents a breakdown or loss of continuity of a fascial closure. This is at most manifested by a bulge in an abdominal wall closure that is detected either visually or by direct palpation. Increased intra-abdominal pressure by coughing or lifting typically makes an incisional hernia more apparent [Figure 1]. Occasionally, patients with large hernias experience difficulty in bending, discomfort, even persisting pain or intermittent intestinal obstruction. On the other hand, most patients with small uncomplicated incisional hernias will be asymptomatic or have only minor or intermittent complaints. As with other hernias, incarceration or strangulation is much more common if the neck of the hernia defect is narrow. The presence of an incisional hernia is typically apparent during clinical examination. Furthermore, ultrasonography and computer tomography can be applied to distinguish hernia defects from other abdominal wall processes as mass lesions representing the source of pain syndromes.

### Mesh materials

**Figure 2:**
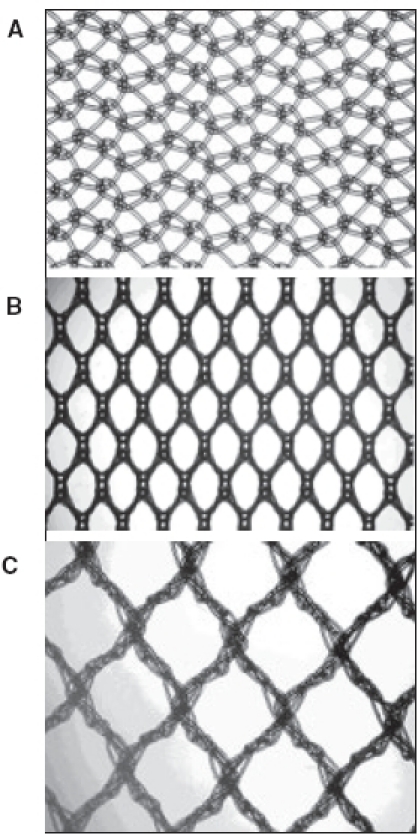
Commonly used mesh materials: A. polypropylene mesh (Atrium), B. polyester mesh (Mersilene), C. material-reduced composite mesh made of polypropylene and absorbable polyglecaprone (UltraPro) original magnification 12.5x

Mesh materials for abdominal wall hernia repair are likely the most common biomaterials implanted in surgical medicine. Since the introduction of synthetic mesh materials into surgery by Usher, our understanding of mesh biology has developed continuously. By now, intensive research in the field of mesh structures, wound-healing properties, tissue response and mesh integration has led to an improvement of hernia repair. In general, the ideal mesh is characterized by economic aspects, functionality and operative handling, sterility or even anti-infective and optimized biocompatibility. Surgical meshes consist of different polymers with an either mono- or multifilament structure. Furthermore, relevant textile parameters in their evaluation are the amount of material, the tensile strength, the bending resistance and the elasticity. Particularly for laparoscopic hernia repair, the pore size and the transparency of meshes play a pivotal role. Based on their polymer structures, the most frequently used materials in hernia repair can be grouped into the following four candidates [Figure 2].

### Polytetrafluoroethylene (PTFE: Gore-Tex)

Meshes made of polytetrafluoroethylene are characterized by a very small pore size (1–6 µm) and therefore react like a foil without tissue ingrowth. Although providing sufficient mechanical stability, until now, there is little information about the long-term degradation of PTFE meshes. Because of a low affinity for adhesions, the PTFE mesh is probably the first choice for an intraperitoneal position of the prosthesis (laparoscopic repair). Due to the small pore size however, the persistence of bacteria is promoted, thus leading to higher infection rates as compared to other materials. Therefore a removal of the prosthesis in case of infections is necessary. Another disadvantage of these meshes is its high cost.

### Polyethylene (Mersilene, Parietex)

Polyethylene meshes show high mechanical stability and provoke only a slight adhesion formation. With the construction of multifilaments, the low-weight Mersilene mesh shows a reasonable flexibility, whereas the high-weight multifilament Parietex mesh is characterized by a high bending resistance. One problem of polyethylene meshes is their degradation, which leads to a reduced mechanical stability after 10 years. Moreover, this hydrolytic disintegration has been shown to be catalyzed by a persisting infection. Finally, it has also been reported that patients with polyethylene mesh implants have higher incidences of wound-healing complications, fistula and seroma formation and higher incidences of hernia recurrence as compared to polypropylene meshes. To summarize, due to the loss of stability and the reported mesh-related complications, polyethylene meshes nowadays do not seem to be fully suitable for a permanent reinforcement of the abdominal wall.

### Polypropylene (Marlex, Prolene, Atrium, SurgiPro)

Polypropylene meshes show good mechanical stability and reasonable elasticity. Until now, a long-term degradation similar to polyethylene meshes has not been reported. In contrast to PTFE meshes, polypropylene meshes are less susceptible to infections and even might be left *in situ* in case of an infection. Polypropylene meshes are often constructed with monofilaments with a small pore size and a heavyweight character. After an initially pronounced inflammatory tissue reaction, most materials provoke a relatively long-lasting and ‘mild’ chronic foreign body reaction. Because of postoperative seroma formation in 14.5–45% of implantations, the application of drainages for 3–7 days is recommended.

### Material-reduced meshes: Polypropylene and polyglactin (Vypro I/II), polypropylene and polyglecaprone (UltraPro)

In order to avoid the disadvantages of high amounts of material, like the strong foreign body reaction or the subsequent increase of abdominal wall restriction, a material reduction of polypropylene meshes was discussed. Furthermore, investigations about the tensile strength of the abdominal wall with a maximum of 16 N/cm have led to the assumption that common polypropylene meshes are oversized. With the development of the Vypro mesh, the first low-weight mesh with a reduction of 70% of polypropylene was constructed. At this stage, polyglactin 910 filaments were added to polypropylene multifilaments to increase its bending resistance and improve intraoperative handling. Because of its large pore size, this ‘light’ mesh has been shown to preserve a high level of elasticity even when incorporated into scar tissue. Additionally, as shown by Klinge *et al,* the tissue response to implanted Vypro meshes is characterized by a significant reduction of inflammation and fibrosis, thereby leading to a more physiological integration with formation of a scar-net instead of a stiff scare plate. Accordingly, less pain, mesh awareness and stiff abdomen were noted subjectively in these patients. The absorbable polyglactin 910 part disappears completely after 3 months of implantation, leaving slightly waved polypropylene filaments. However, despite clinical advantages, there is still concern about the use of multifilaments with regard to a possible potentiation of infection through the interstices of the braided structure. Klinge *et al* showed that the increased surface area promotes the persistence of bacteria in the implant bed. This might explain the development of mesh-related infections even after a delay of several months or even years, favoring the use of monofilament materials. Therefore a new monofilament low-weight composite mesh (UltraPro) was constructed made of nonabsorbable polypropylene monofilaments and supplemented with absorbable monofilament polyglecaprone 25 (Monocryl) threads. This mesh was found to be feasible, with no additional short-term mesh-related complications in the experimental model and no negative side effect on biocompatibility.

## REQUIREMENTS DEMANDED FOR SURGICAL MESH

To conclude, the task of a surgical mesh is a permanent reinforcement of the abdominal wall. Its strength and elasticity should be adapted to the highest physiologically required forces and therefore to a maximum load of the intra-abdominal pressure of 100–150 mmHg. Except for abdominal wall replacement, where a tensile strength of 32 N/cm should be provided, the reinforcement of the abdominal wall with a sufficient fascial closure in front as thrust bearing requires a strength of 16 N/cm. With respect to these physiologically required forces, the values of conventional mesh materials are often disproportional. As the inflammatory tissue reaction correlates with the amount of the incorporated material, the lowest possible weight combined with a high elasticity is recommended. Differences in pore size have been suggested as another explanation for differences in the inflammatory reaction to surgical meshes. Beets *et al* found an increased foreign body reaction with polypropylene meshes with smaller pores. In this context, Klinge *et al* postulated an impaired fluid transport through small pores to be responsible for an accentuated tissue response. A pore size lower than 800–600 µm results in a fibrotic tissue response similar to a ‘bridging reaction’ between the pores, thus rather leading to a scar plate than a scar net. We therefore recommend the use of large-pored mesh materials. The use of monofilament meshes should further reduce bacteria adherence and mesh infections. In future, the development of mesh materials should improve both its biocompatibility and - as a scaffold - the patients' wound-healing and regenerative properties. Meshes with an even biological activity could reduce recurrence rates in hernia patients possibly suffering a connective tissue disorder.

### Sublay mesh technique

Apart from surgical repair, other than the use of an abdominal support in the frail and elderly, there is no alternative treatment for incisional hernia. Hernia repair should be considered early because of the tendency for the defect to increase in size and impair quality of life. Apart from previous technical faults or traumatic defects of the abdominal wall, the surgical technique should routinely include the use of mesh. The only exception may be small defects of less than 3 cm, which can be closed by a continuous nonabsorbable suture repair (a suture length/wound length ratio of 4:1), but such an approach is tailored to the individual situation. In the case of a giant hernia or obesity, preoperative improvement of respiratory function and reasonable weight reduction[15] should be encouraged. Additionally, the skin should be in optimal condition to minimize the risk of infection. Preoperative bowel preparation and perioperative antibiotics are advisable. In principle, flat mesh is placed in a prefascial, retromuscular position to reinforce the fascia closure and form an extended mesh-scar compound. After excising the entire skin scar, the hernia sac is prepared down to the margins of the fascia. The sac is then opened and local adhesiolysis eases the complete opening of the previous incision. For midline incisions, the retromuscular space behind the rectus muscles and in front of the posterior rectus sheath (prefascial) is bluntly dissected. The neuro-vascular bundles at the lateral part should be preserved as carefully as possible. At the cranial margin, the posterior sheath is incised on both sides parallel to the linea alba. A triangle of preperitoneal fat with separating fascial margins becomes apparent.[16] To achieve a sufficient overlap (at least 5 cm), preparation continues far behind the xiphoid. Similar preparation is needed at the caudal margin of the fascial incision, where the disappearing posterior rectus sheath favors dissection in the preperitoneal space below the arcuate line. Finally, the mesh must be placed behind the pubic bone in front of the bladder. It is advisable to complete the circular preparation of the preperitoneal mesh placement before closing the peritoneum to avoid damage to closely attached bowel or organs during the dissection. A major task is the prevention of direct contact between the bowel and the mesh prosthesis to avoid dense adhesions or late fistulas. Thus, the peritoneum must be carefully closed by continuous absorbable suture. A further interposition of omentum might be helpful, especially in cases of peritoneal defects. After careful control for bleeding, the mesh is trimmed to fit the specific dimensions of the defect to be treated. Usually, implants have a width of 12 to 14 cm and a length of 20 to 35 cm. Respecting the physiologic elasticity of the abdominal muscle fibers, the mesh should feature its main elasticity in a vertical direction. This ensures adaptation to the physiological stretchability of the abdominal wall and prevents craniocaudal shrinkage by mesh deformation. An overall overlap of at least 5 cm in all directions is mandatory. To prevent early dislocation, the unfolded mesh is fixed circularly to the posterior rectus sheath and the peritoneal sac below the arcuate line respectively. It remains controversial as to whether the use of nonabsorbable sutures is absolutely indicated. During fascia closure, wrinkling of the mesh should be avoided. After placing drains in the retromuscular space, the anterior fascia is closed by nonabsorbable continuous suture respecting a 4:1 ratio for suture/wound length. Working as a thrust bearing and preventing early strain to the mesh, closure of the fascia is imperative. If closure of the anterior fascia would occur with undue tension, relaxing incisions in the anterior rectus sheath or an additional Ramirez component separation is sometimes required. Skin closure follows as usual. Postoperative care is mainly directed to the ‘control of wound’ problems. Because the mesh is assumed to be integrated, mobility restriction is required for no longer than 1 week. Only the repair of giant hernias sometimes demands prolongation of postoperative artificial respiration until respiratory function has fully recovered.

## RESULTS

Postoperative results are frequently complicated by seroma formation, wound infections, wound discomfort and recurrence. Whereas a sizable seroma is seen in about 30% of the patients, it rarely requires reintervention apart from aspiration. However, there always are a few patients with excessive fluid accumulation around the wound who require surgical intervention and removal of the seroma capsule, which may have persisted for months. Infections may be expected in about 10% of the patients. Usually restricted to the subcutaneous space, they should be treated conservatively as common wound infections. Even if the infection encroaches into the mesh itself, a conservative attempt is justifiable, provided the mesh is porous. Late infections appearing after months or even years are more challenging. They are often combined with complex fistulas including bowel.[17,18] In these cases, preservation of the mesh is likely to fail and sooner or later most of the mesh has to be removed. After a temporary mesh-free closure, any subsequent mesh repair should be performed no sooner than 6 months later. Moderate complaints after incisional hernia repair are quite common, especially in patients with a long history of previous incisions. Fortunately, the development of a ‘stiff abdomen’ is rare, although it sometimes requires a mesh exchange.[19] Whether modern large-pore meshes with preserved elasticity can prevent this unpleasant complication is not yet clear. For patients and surgeons, recurrence is the most concerning complication. Owing to the stability of the implants, mesh ruptures remain rare. Recurrences are mainly at the border of the mesh, indicating insufficient overlap. Of 1,070 incisional hernia repairs performed in our department since 1995, only 77 patients required reoperation for recurrence after mesh repair. The median time to recurrence was 21 months. In 36 patients, the previous incision crossed the linea alba. In 25 cases, the recurrence manifested in the subxiphoidal area. In 3 cases of abdominal wall defect, the recurrent hernia passed through the mesh. Five recurrences of an incisional hernia in the flank appeared at the lateral border of the repair; and in 10 other patients the relapse was located at the caudal margin. Most of those affected (n = 34) underwent a second mesh implantation at a revisional operation and an attempt was made to increase the width of overlap by healthy tissue. The remaining patients received a complete mesh exchange (n = 31), a closure of the circumscriptive defect reinforced by the onlay technique (n = 2), mesh removal and suture repair (n = 5) or a suture closure of the fascial margins due to a complex infection (n = 5).

## CONCLUSIONS

Despite recurrences after mesh implantation, the recently published data are encouraging. These series prove the superiority of mesh compared to simple suture repair [Table 1]. In summary, the use of mesh can reduce the recurrence rate from 40–50% to about 10%.[24,25] Even if this effect represents only a delay in the appearance of a recurrence, it reduces morbidity and the rate of reoperation required for re-recurrence. Perhaps an extensive overlap can prolong this delay for the rest of the patient's life. Lacking valid data, mesh should be positioned behind the abdominal wall muscles (sublay technique) using physiologic abdominal wall pressure for further fixation of the implant. If (and only if) retromuscular placement cannot be achieved, an onlay implant is justified. In the absence of results from randomized trials, closure of the covering fascia is preferred with nonabsorbable suture material because it is a logical step when utilizing nonabsorbable mesh. Extended defects of the abdominal wall where the fascia cannot be closed and the mesh is used to bridge the defect must be reinforced by materials with a tensile strength of >32 N/cm. Further technical pitfalls mainly refer to anatomy but usually can be answered successfully. Mesh explantation is strictly limited to patients with complex infections or a ‘stiff abdomen.’

**Table 1:** Recurrence rates of mesh repair for incisional hernia

Study	Year	No.	Recurrence rate %
Basoglu[17]	2004	264	6.4
Langer[20]	2003	155	14
Leber[18]	1998	119	14.0
Schumpelick[21]	1999	81	5.0
Stoppa[22]	1999	751	6.0
Liakakos[23]	1994	102	6.0
Usher[24]	1962	156	10.2
Burger[10]	2004	84	32.0
